# Niche partitioning in the *Rimicaris exoculata* holobiont: the case of the first symbiotic *Zetaproteobacteria*

**DOI:** 10.1186/s40168-021-01045-6

**Published:** 2021-04-12

**Authors:** Marie-Anne Cambon-Bonavita, Johanne Aubé, Valérie Cueff-Gauchard, Julie Reveillaud

**Affiliations:** 1 Univ Brest, CNRS, IFREMER, Laboratoire de Microbiologie des Environnements Extrêmes, 29280 Plouzané, France; 2 MIVEGEC, Univ. Montpellier, INRAe, CNRS, IRD, Montpellier, France

**Keywords:** Niche partitioning, *Rimicaris*, Metagenome-assembled genomes, *Zetaproteobacteria*, Holobiont, Hydrothermal vents

## Abstract

**Background:**

Free-living and symbiotic chemosynthetic microbial communities support primary production and higher trophic levels in deep-sea hydrothermal vents. The shrimp *Rimicaris exoculata*, which dominates animal communities along the Mid-Atlantic Ridge, houses a complex bacterial community in its enlarged cephalothorax. The dominant bacteria present are from the taxonomic groups *Campylobacteria*, *Desulfobulbia* (formerly *Deltaproteobacteria*), *Alphaproteobacteria*, *Gammaproteobacteria*, and some recently discovered iron oxyhydroxide-coated *Zetaproteobacteria*. This epibiotic consortium uses iron, sulfide, methane, and hydrogen as energy sources. Here, we generated shotgun metagenomes from *Rimicaris exoculata* cephalothoracic epibiotic communities to reconstruct and investigate symbiotic genomes. We collected specimens from three geochemically contrasted vent fields, TAG, Rainbow, and Snake Pit, to unravel the specificity, variability, and adaptation of *Rimicaris*–microbe associations.

**Results:**

Our data enabled us to reconstruct 49 metagenome-assembled genomes (MAGs) from the TAG and Rainbow vent fields, including 16 with more than 90% completion and less than 5% contamination based on single copy core genes. These MAGs belonged to the dominant *Campylobacteria*, *Desulfobulbia*, *Thiotrichaceae*, and some novel candidate phyla radiation (CPR) lineages. In addition, most importantly, two MAGs in our collection were affiliated to *Zetaproteobacteria* and had no close relatives (average nucleotide identity ANI < 77% with the closest relative *Ghiorsea bivora* isolated from TAG, and 88% with each other), suggesting potential novel species. Genes for Calvin-Benson Bassham (CBB) carbon fixation, iron, and sulfur oxidation, as well as nitrate reduction, occurred in both MAGs. However, genes for hydrogen oxidation and multicopper oxidases occurred in one MAG only, suggesting shared and specific potential functions for these two novel *Zetaproteobacteria* symbiotic lineages. Overall, we observed highly similar symbionts co-existing in a single shrimp at both the basaltic TAG and ultramafic Rainbow vent sites. Nevertheless, further examination of the seeming functional redundancy among these epibionts revealed important differences.

**Conclusion:**

These data highlight microniche partitioning in the *Rimicaris* holobiont and support recent studies showing that functional diversity enables multiple symbiont strains to coexist in animals colonizing hydrothermal vents.

**Video Abstract**

**Supplementary Information:**

The online version contains supplementary material available at 10.1186/s40168-021-01045-6.

## Background

Life in deep-sea hydrothermal vent ecosystems is sustained by both free-living and symbiotic microbial chemosynthetic primary production [1]. The shrimp *Rimicaris exoculata* dominates the faunal communities of several hydrothermal sites along the Mid-Atlantic Ridge (MAR) [2–4]. This species lives in dense aggregates in quite a warm part of the hydrothermal environment, 3–25°C, with nearly neutral pH [5], and slightly lower oxygen content than deep seawater [6]. It can be retrieved from geochemically contrasting environments. For example, hydrothermal vents such as those of Snake Pit and TAG, located at 3460 m depth, have developed on basaltic rocks, while Rainbow, located at 2400 m depth, is on ultramafic rock. Fluids emitted by basaltic sites are mainly enriched in sulfur and minerals [7]. In contrast, ultramafic fluids are relatively depleted in sulfur but enriched in methane, hydrogen, and ferrous iron [7], which may select for specific microbial chemoautotrophic communities. Whatever the site of sampling, adult *Rimicaris* harbor complex bacterial communities, one located in their hypertrophied cephalothoracic cavity and a second in their digestive system, where long microbial filaments have been identified whose role remains unknown [8–10]. The cephalothoracic cavity microbiota was first described as a single campylobacterial lineage (formerly called *Epsilonproteobacteria* [11–13])*.* It nevertheless proved to be much more diverse, including a secondary dominant lineage, *Gammaproteobacteria* [14–17], but also, to a lesser extent, *Alpha* and *Deltaproteobacteria* (renamed *Desulfobulbia*) [14, 18–20] and the more recently hypothesized *Zetaproteobacteria* [21]*.* In vivo experiments provided evidence for direct nutritional transfers from bacteria to the host across the cephalothoracic chamber integument and, therefore, indicating a true trophic association [22]. In addition, in silico and microscopy analyses suggest that four potential metabolic pathways (iron, sulfide, methane, and hydrogen oxidation) may co-occur within this community [14, 15, 18, 21]. This diversity of metabolisms indicates that the epibiotic community associated with *R. exoculata* is highly plastic, providing a potential adaptive advantage for the shrimp thriving in highly dynamic hydrothermal mixing zones. This diverse bacterial consortium could explain the clear success of the holobiont (*i.e.*, the animal in interaction with its symbiotic partners) in colonizing these geochemically contrasted hydrothermal vents all along the Mid-Atlantic Ridge [4]. However, although previous studies identified diverse microbial populations, authors focused on dominant metabolisms and community members, leaving knowledge gaps regarding the overall functioning of co-occurring symbionts, some of which have been very little studied. Briefly, Jan and colleagues [21] described the dominant lineages, *Campylobacteria* and *Gammaproteobacteria*, and made a new hypothesis on the under-recovered *Zetaproteobacteria*. They focused on carbon fixation pathways (rTCA and CBB cycles for *Campylobacteria* and *Gamma*/*Zetaproteobacteria*, respectively), sulfur, hydrogen and nitrogen cycles, stress response, and interactions with the host. Still, authors identified taxobins as submetagenomes and did not undertake genome reconstruction [21]. Recently, Jian et al. [19] focused on *Desulfobulbia* and described a novel species, Candidatus *Desulfobulbus rimicarensis*, with specific symbiotic traits. However, the functional capacities and evolutionary relationships of the remaining epibionts, notably the *Zetaproteobacteria*, under-recovered compared with their free-living counterparts, remain unknown.

Biotic iron oxidation reactions were first hypothesized to occur in the *Rimicaris* cephalothoracic cavity based on observations of iron oxyhydroxide particles embedding microbial cells [6]. These mineral deposits are located in the upper part of the cavity where the outgoing seawater flow is expulsed. Here, they may be under microaerophilic conditions and partially enriched in carbon dioxide due to shrimp respiration. Two subsequent studies showed these iron particles have a stable mineral composition, with increasing deposits through the molt cycle, giving shrimps a rusty color by the end [23, 24]. An in vivo experiment also showed that chemoautotrophy was fueled by Fe^2+^ supply as the sole electron donor [22]. Still, only a very few genes affiliated to potential iron oxidizers, namely *Zetaproteobacteria*, have been identified in studies using molecular approaches [21], leading to major gaps in knowledge regarding potential symbiotic iron oxidation. It is possible that this coating with iron oxyhydroxides, closely attached to the bacterial cells [23, 24], impaired genomic DNA extraction from these specific epibionts.

In addition to these physical constraints, reconstructing and separating highly similar genomes from metagenomes, notably from complex host-associated microbial communities, remained a technical challenge until recently. The presence of closely related strains can increase assembly breaks, resulting in short orphan contigs that cannot be grouped together nor further analyzed. In addition, unlike for symbionts constrained in a specific organ such as tubeworm trophosomes [25], and despite precautions in the DNA extraction protocols, host contamination is unavoidable and can result in a high number of host-associated contiguous sequences. Therefore, efforts to understand the functioning of the epibiont community are still in their infancy. In particular, it remains unknown whether the highly diverse *Rimicaris* epibiont community is composed of specialists adapted to microniches that possibly complement or interact with each other through syntrophy, or rather composed of generalists able to perform a wide range of functions depending on the environmental conditions.

Here, we improved DNA extraction procedures to maximize DNA recovery from iron/mineral-embedded bacteria, followed by shotgun metagenomics and advanced binning strategies to reconstruct novel *Rimicaris exoculata* symbiotic genomes. We collected specimens from the three geochemically contrasted hydrothermal vents TAG, Snake Pit, and Rainbow to investigate functional diversity and assess potential niche differentiation at the genome level within and between sites. We aimed to decipher the functioning of the *Rimicaris* epibiont community as a whole, to better understand animal–epibiont interactions at a fine genomic scale and, for the first time, propose an overview of the capabilities of *Zetaproteobacteria* as symbionts.

## Methods

### Sample collection

**Fig. 1:**
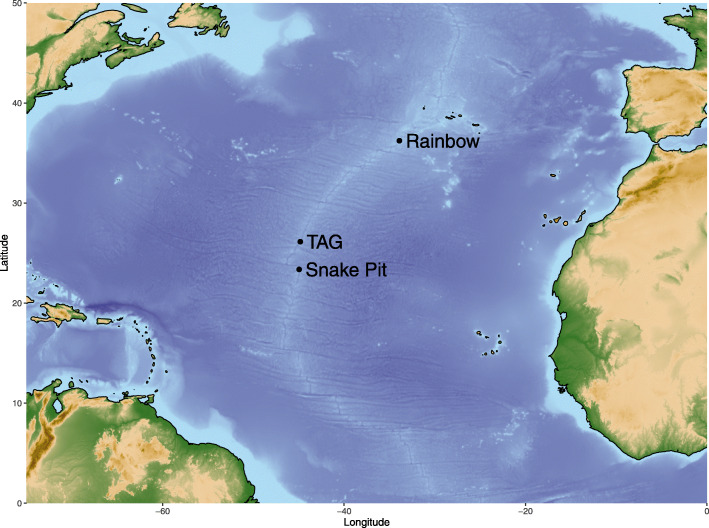
Geographic locations of Mid-Atlantic Ridge hydrothermal vent sites Rainbow, TAG, and Snake Pit where *Rimicaris* specimens were sampled

We collected *Rimicaris exoculata* specimens using a suction sampler manipulated by the remote operated vehicle (ROV) Victor 6000, controlled from on board the research vessel *Pourquoi pas?* Samples were taken from three hydrothermal sites on the Mid-Atlantic Ridge (Fig. 1): Rainbow (36° 13.760′ N, 33° 54.170′ W, 2292m depth) during the MoMAR (Leg2, August 25 to September 15, 2008, 10.17600/8010140) and BioBaz cruises (August 02 to 21, 2013, 10.17600/13030030), TAG (26° 8.237′ N, 44° 49.563′ W, 3625 m depth) and Snake Pit (23° 22.140′ N, 44° 57.054′ W, 3495 m depth) during the BICOSE cruise (January 11 to February 10, 2014, 10.17600/14000100), respectively. We rapidly froze the BioBaz and BICOSE samples at − 80°C on board for later genomic DNA extraction. We dissected branchiostegites and scaphognathites of some MoMAR specimens directly onboard under sterile conditions, fixed them for three hours in 3% formaldehyde seawater solution, and stored them frozen in PBS/Ethanol 1:1 until further use for FISH experiments.

### DNA extraction

We performed sterile dissections in the lab on thawed samples before total genomic DNA extraction of the six *Rimicaris* cephalothoracic appendages (2 branchiostegites + 2 scaphognathites + 2 exopodites). As mentioned above, symbionts have previously been found embedded in iron particles, which may impair DNA extraction. To maximize DNA recovery, we chose specimens in the middle of their molt cycles, to avoid over embedding in minerals, but with high levels of microbial colonization according to earlier color-based identification [23]. Then, we aseptically crushed each sample for few minutes in a sterile mortar in iced lysis buffer to release as many bacteria as possible from the shrimp cephalothorax. We then used a vortex step of 5 min on the crushed samples to help separate bacteria from minerals. The DNA was then extracted using the Nucleospin Soil extraction kit (Macherey-Nagel) following the manufacturer’s instructions.

### Microbial community DNA preparation and sequencing

We analyzed six *Rimicaris* individuals (hereafter referred to as RE5 and RE6 from TAG, RE12, and RE13, from Rainbow, and RE3 and RE7 from Snake Pit) by shotgun sequencing of total community DNA. We quantified DNA using a NanoDrop 1000. We sheared DNA to 175 bp using a Covaris S-series sonicator and completed metagenomic library construction using the Ovation Ultralow Library DR multiplex system (Nugen) following the manufacturer’s instructions. We performed metagenomic sequencing on an Illumina HiSeq 1000 and a NextSeq at the W.M. Keck sequencing facility at the Marine Biological Laboratory (MBL, MA, USA). All libraries consisted of paired-end data, with a 30-bp overlap.

### Metagenomic analysis

We trimmed adaptors using bbduk from bbmap 38.22 [26] after which we processed reads using a snakemake [27] workflow implemented in anvi’o v5 (http://merenlab.org/2018/07/09/anvio-snakemake-workflows/) [28]. Briefly, we used illumina-utils v1.4 [29] with the “iu-filter-quality-minoche” program with default parameters for sequence quality filtering. We co-assembled two metagenomic datasets per site (that is, RE5 and RE6 for TAG; RE12 and RE13 for Rainbow; and RE3 and RE7 for Snake Pit) using Megahit 1.1.3 [30] and the --metasensitive mode, discarding contigs smaller than 1000 bp. We performed read recruitment analyses with Bowtie v2.3.4 [31]. We used the option “all-against-all” to map reads from each of the six individual samples onto each of the three co-assemblies. The details and a scheme of the bioinformatics pipeline are provided in Additional File 1.

To estimate symbiont relative abundance, we retrieved the raw counts mapping to each MAG using samtools view [32]. We normalized data within and between samples with Gene length corrected Trimmed Mean of M-values (GeTMM, [33]) using genome length instead of gene length. We used DESeq2 [34] for differential MAG abundance analysis between sites. Differentially abundant MAGs with adjusted *p*-value of 0.01 (padj < 0.01) and absolute log2 fold change of 2 were regarded as significant in this study.

For phylogenomic analyses, we searched for and aligned 120 bacterial marker genes of the MAGs using the identity and align commands of GTDB-Tk v1.1.0 [35]. We filtered closely related GTDB taxa identified with the “classify_wf” workflow with the taxa-filter option during the alignment step. We trimmed multiple sequence alignments using trimAl v1.4.1 [36] with the setting “-gt 0.5” to remove positions with gaps in more than 50% of sequences. We reconstructed a Maximum Likelihood (ML) phylogenetic tree using IQ-TREE v1.6.12 [37] with the “WAG” general matrix model [38] and 1000 bootstrap replicates that we visualized using anvi’o and FigTree v1.4.4. Finally, we made a finer GTDB reference taxa filtration after a first visualization, providing a better representation of our MAG genera. For this purpose, we removed references from distant families and added some from the closest genera or families in a supervised manner.

We calculated average nucleotide identity (ANI) online using the ANI calculator (https://www.ezbiocloud.net/tools/ani) [39]. As for functional analyses, we extracted KO assignations from the anvi’o database for each MAG and used the KEGG Decoder v 1.0.8.2 (www.github.com/bjtully/BioData/tree/master/KEGGDecoder) [40] to determine the completeness of various metabolic pathways based on a key set of genes. In addition, we used the RAST platform to provide a more detailed analysis of the different *Zetaproteobacteria* genomes (from this study and their free-living counterparts). The schematic representation of the predicted metabolic potentials within *Zetaproteobacteria* MAGs was constructed based on the KEGG annotations and the Reconstruct Pathway tool (https://www.genome.jp/kegg/tool/map_pathway.html). To explore the taxonomic composition of each samples based on the small-subunit rRNA, the quality-filtered reads were analyzed using the phyloFlash v3.4 pipeline [41] with the option “almost everything” and the SILVA database release 138.1 [42].

### Code and data availability

The metagenome raw reads are available in the European Nucleotide Archive under Bioproject Accession Number PRJEB37577. We also made the FASTA files available for individual metagenomic co-assemblies and for the 49 MAGs at DOI (10.12770/4186eeef-32b1-4ffb-9a40-61a02a3852b7), as well as the anvi’o merged profile databases for each co-assembly and MAG. The URL https://gitlab.ifremer.fr/rimicaris/rimicaris-exoculata-cephalothoracic-epibionts-metagenomes provides access to a detailed reproducible bioinformatics workflow for all the computational analyses.

### Fluorescence in situ hybridization procedures

We carried out FISH procedures as described by Duran et al. [9]. Briefly, we hybridized 0.6-μm transverse sections using the Zeta123-Cy5 probe (5′-ACTGATGGGCAGGTAACCACG-3′, [43]) directed toward *Zetaproteobacteria*, and the EPSI549-Cy3 probe (5′-CAGTGATTCCGAGTAACG-3′, [44]) directed toward *Campylobacteria*, together with DAPI staining for cell nucleus observation. Hybridization parameters were set at 35°C and 55% formamide as in Jan et al. [21]. Nonsense probes were tested but gave no signal. For observations, we used an Apotome AxioImager Z2 equipped with a Colibri LED system (Zeiss, Göttingen).

## Results and discussion

In this study, we used metagenomic data of *Rimicaris exoculata* shrimp epibiont communities from three contrasting hydrothermal sites along the Mid-Atlantic Ridge to reconstruct bacterial genomes and examine functional potential at the genome-resolved scale.

### The reconstruction of 49 *Rimicaris-*associated MAGs

Shotgun sequencing of total community DNA recovered from the cephalothorax of six *R. exoculata* individuals (RE3, RE5, RE6, RE7, RE12, RE13) yielded 22–119 million paired-end sequences. Metagenomic assembly of each site yielded 34K–64K contigs longer than 1 kbp, which recruited 14–92% of the raw sequencing reads. Additional File 2 provides the statistics on the raw number of reads, quality trimming, filtering, assembly, and recruitment results for each sample.

We clustered contigs across the six *Rimicaris* specimens with respect to their sequence composition signatures and differential coverage patterns. This binning approach allowed us to efficiently segregate the metagenomic assembly into 29 genome bins for Rainbow and 21 for TAG. We retained bins with more than 2 Mbp or more than 60% completion and less than 10% contamination based on the single occurrence of 71 single-copy core genes (SCG) that represent a modified version of the HMM profiles published by Lee [45] implemented in anvi’o [28]. We were unable to reconstruct bacterial MAGs for Snake Pit using these criteria, due to a much lower sequencing depth for shrimp samples from this site compared with the Rainbow and TAG ones. Chemically contrasting site Snake Pit however proved informative because it provided differential coverage values that improved the binning strategy.

**Fig. 2:**
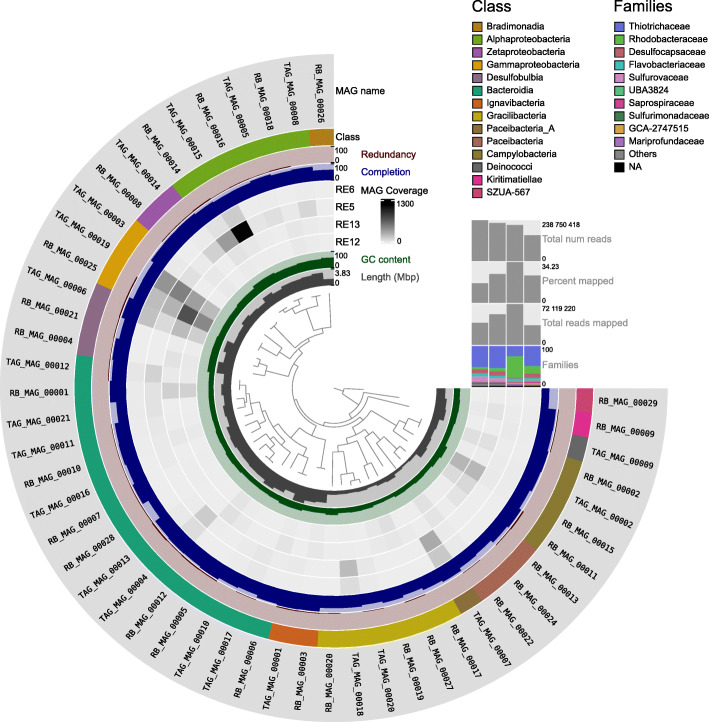
Static image from the anvi’o interactive display for the *Rimicaris* datasets with the 49 genome bins retained for this study. From inner to outer layers: phylogenomic tree based on concatenated marker proteins according to GTDB-Tk, length layer (shows the actual length of a genome), auxiliary layer with information about contigs stored in the contig database (GC-content), four view layers with information about MAGs across samples stored in the profile database (mean coverage), completion, redundancy, genome class based on GTDB-Tk, and bacterial genome bin layers. The horizontal layers show genome bin taxonomy based on GTDB-Tk for the ten most abundant families, total number of reads mapped and corresponding mapping percentage of reads, and total number of reads for each sample. Relative abundances of families are noted as the percentages of reads recruited to the bins for each sample

Dereplication (an alignment fraction between genomes of both 0.10 and 0.75%) generated a single cluster containing TAG_MAG_00006 and RB_MAG_00023 *Desulfocapsa* (*Desulfocapsaceae*), with lower scores for the latter, which was then excluded. This resulted in a final collection of 49 MAGs to be further analyzed (Fig. 2). We nevertheless note that the 49 MAGs reconstructed and described herein represent only part of the *Rimicaris* cephalothorax diversity and that some remaining groups might be misrepresented given the fact that strain diversity can result in assembly breaks and therefore impair binning and recruitment analyses [46]. MAG collections for TAG and Rainbow, with estimates of completion and redundancy, total bin length, and taxonomic affiliation determined by GTDB-Tk, are provided in Additional File 3. We used read mapping against these 49 newly reconstructed genomes and normalization with GeTMM to estimate symbiont relative abundance within and between samples (Additional File 4). We used DESeq2 to compare the differential abundance of the MAGs between sites (Additional File 5 and 6). We hereafter focus our distribution analyses according to vent origin on the most abundant genome bins.

### Abundance of *Campylobacteria*, *Desulfobulbia* (formerly *Deltaproteobacteria*), *Gammaproteobacteria*, *Alphaproteobacteria*, and newly recovered candidate phyla radiation (CPR) lineages

We observed a small number of symbionts that seemed to dominate at the different hydrothermal vent sites (Additional Files 4, 5, 6). The two most abundant MAGs at ultramafic Rainbow, RB_MAG_00025 *Gammaproteobacteria* (*Thiotrichaceae* family) and RB_MAG_00022 *Patescibacteria* (GCA-2747955 family), were not differentially abundant at any site. On the other hand, the third most abundant MAG at Rainbow RB_MAG_00015, *Campylobacteria* (*Sulfurovaceae* family), was significantly more abundant at Rainbow than at TAG (log2FoldChange>3). Similarly, we observed RB_MAG_00011, *Campylobacteria* (*Sulfurimonadaceae* family), in much higher abundance in the shrimps from Rainbow than TAG (log2FoldChange>7). Both lineages were retrieved from moderate temperature fluids with quite elevated sulfide concentration and in the presence of oxygen. *Sulfurovaceae* are well known to occur as symbionts associated with hydrothermal fauna, but this is less the case for *Sulfurimonadaceae*, whose genomes have been recently sequenced from *Alviniconcha* snails colonizing almost the same biotope as *Rimicaris* in Pacific hydrothermal vents [47]. The seemingly absence of dominant *Campylobacteria* MAGs at TAG, although they had been observed in previous studies [48], could be due to the presence of closely related *Campylobacter* strains impairing genome reconstruction of these populations at this site. Diverse *Campylobacter* were actually observed based on small-subunit rRNA reconstruction using phyloFlash (Additional File 7). The ultramafic Rainbow fluids partly depleted in sulfur and enriched in hydrogen may be more selective for a few *Campylobacter* lineages able to cope with hydrogen, while many closely related species could be present at TAG. Deep-sea vent *Campylobacteria* have also been shown to lack many DNA-repair genes, which could lead to numerous recombination, mutation, gene loss, and horizontal gene transfer events increasing microdiversity and conferring genomic plasticity on these taxa [49].

On the contrary, TAG most abundant MAGs TAG_MAG_00015 and RB_MAG_00014, *Alphaproteobacteria Marinosulfonomonas* sp. (*Rhodobacteraceae* family) were significantly more abundant at TAG than Rainbow (log2FoldChange =−5.5 and −3.13, respectively). In addition, abundant TAG_MAG_00007 (*Candidatus Patescibacteria* phylum, *Paceibacteria* class, from the candidate phyla radiation (CPR)) [50] showed a higher abundance at TAG as compared to Rainbow (log2FoldChange =−9.7). The latter contrasted with MAG RB_MAG_00022 from the same phylum and class, which did not show any significant differential abundance at Rainbow and TAG shrimps. TAG_MAG_00018, from the same phylum (*Gracilibacteria* class), was also significantly more abundant at TAG than Rainbow (log2FoldChange=−2.8). The predominance of these groups in the *Rimicaris* holobiont, associated with small genome size for TAG_MAG_00007, TAG_MAG_00018, and RB_MAG_00022 (1,201,735 bp, 841,694 bp, and 624,325 bp, respectively), adds to recent studies showing a wide distribution of CPR organisms. These lineages are often reported in association with abundant autotrophic taxa involved in nitrogen, sulfur, and iron cycling [51, 52] as well as arsenic in contaminated sediments [53].

We also observed several MAGs which abundance was not significantly different at both sites (abs(log2FoldChange)<2) like TAG_MAG_00006, *Desulfobulbia* (*Desulfocapsaceae* family), and RB_MAG_00001, *Bacteroidia* (*Flavobacteriaceae family)*. Like for its counterpart RB_MAG_00025, TAG_MAG_00019 *Gammaproteobacteria* (*Thiotrichaceae* family) was not differentially abundant at both Rainbow and TAG shrimps.

These data provide further evidence for the occurrence of similar genomes in each host individual regardless of the site of sampling, yet with varying abundance partly reflecting contrasting environmental conditions. This may indicate that the cephalothoracic cavity, a nearly closed environment, protects symbionts from steep environmental modifications, leading to an overall similar symbiotic community whatever the site. Alternatively, a second hypothesis, not exclusive from the previous one, is that the shrimp have a pool of diverse symbiotic bacteria that can express their potential according to the chemical signature, thus explaining the colonization success of this holobiont.

### Dual *Zetaproteobacteria* symbiosis in *Rimicaris: Candidatus Ghiorsea rimicarensis* and *Candidatus Ghiorsea crypta*

Iron-oxidizing *Zetaproteobacteria Mariprofundus ferrooxydans* PV-1 and JV-1 isolates were first described from the Lō’ihi hydrothermal systems in Hawaii [54, 55]. *Zetaproteobacteria* have since been described from very distinct habitats showing high ferrous iron concentrations, including coastal sediments, steel corrosion biofilms, and saline terrestrial springs [56, 57]. They are also reported at the MAR hydrothermal sites of the present study: TAG, Rainbow, and Snake Pit, where they dominate iron rich microbial mat communities [58]. These specialized, yet diverse, bacteria are well adapted to microaerophilic growth on Fe(II) and play an important role in the biogeochemical iron cycle within these diverse ecosystems, where abiotic iron oxidation was first thought to be the rule [43, 59]. Two distinct *Zetaproteobacteria* symbiotic genomes with high completeness, TAG_MAG_00014 and RB_MAG_00008, were recovered for the first time at both TAG and Rainbow sites. *Zetaproteobacteria* TAG_MAG_00014 was significantly more represented at TAG (log2FoldChange=−5.36, Additional File 5). However, *Zetaproteobacteria* RB_MAG_00008 was not significantly more abundant at Rainbow or TAG (abs(log2 Fold Change)<2). Differential abundance for the two lineages could suggest that specific *Zetaproteobacteria* lineages dominate at distinct or within a single hydrothermal vent site, depending on the environmental conditions. Surprisingly, the number of *Zetaproteobacteria* was higher at TAG, where *G. bivora* was also isolated [60], than at the iron-richer Rainbow site. This may be due to more robust iron incrustations on Rainbow specimens, despite the use of a thorough DNA extraction protocol.

**Fig. 3:**
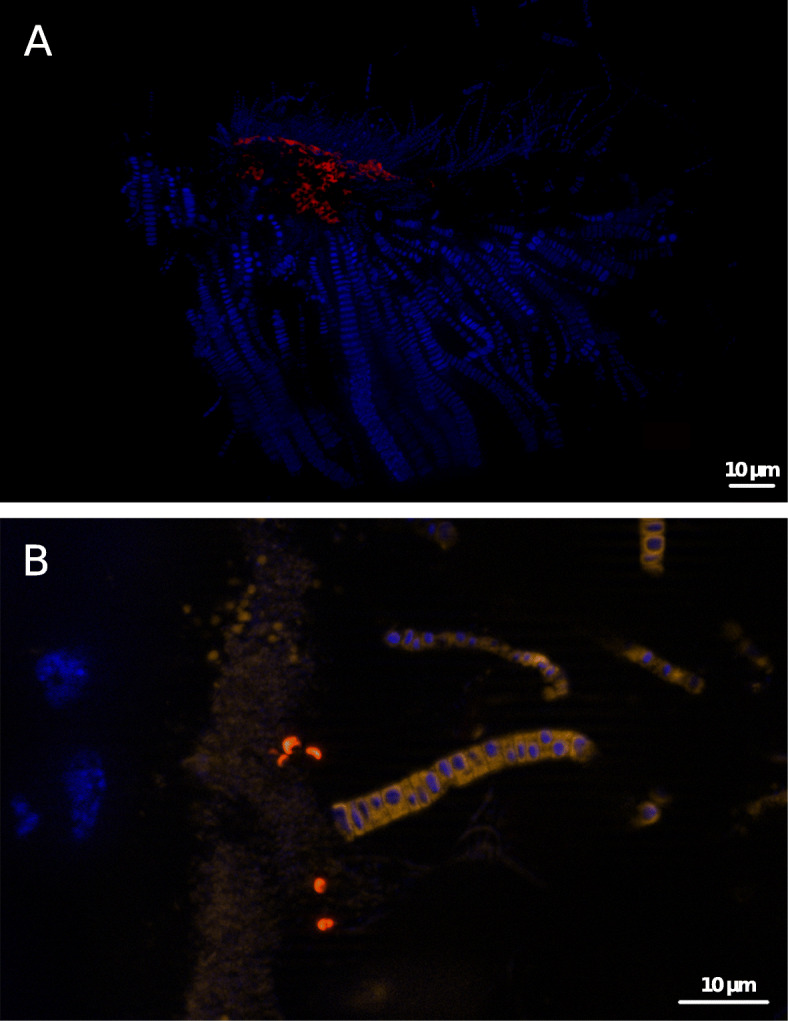
FISH observations of a Rainbow *R. exoculata* branchiostegite with epibionts. All cells are labeled with DAPI (blue). *Zetaproteobacteria* are hybridized with the Zeta123 probe (red) [43]. **a** *Zetaproteobacteria* are closely attached to the cuticle. Dominant large filamentous bacteria (*Campylobacteria*) and thinner *Gammaproteobacteria* filaments can be seen. **b** Co-hybridization of filamentous *Campylobacteria* with the EPSI 549 probe (orange) [44] and small *Zetaproteobacteria* rods (red), highlighting the co-occurrence of both lineages

Fluorescence in situ hybridization revealed *Zetaproteobacteria* in previously sampled Rainbow *R. exoculata* individuals [21], yet their genomic potential and potential microdiversity remained hidden. Here, we confirmed these findings using a similar FISH procedure, showing cells as small, curved rods, closely attached to the host cuticle under the long filamentous *Campylobacteria* symbionts (Fig. 3a, b). These observations are in agreement with previous TEM observations [24]. Unfortunately, we were able to retrieve one 16S rRNA gene for *Zetaproteobacteria* TAG_MAG_00014 only, and not for RB_MAG_00008. A blast analysis between the 16S rRNA gene full-length sequence extracted from TAG_MAG_0014 and the ones retrieved using PhyloFlash from TAG (RE5 and RE6) and Rainbow (RE12 and RE13) showed 100% and 97.7% identity, respectively (Additional File 8). Overall, data did not allow designing specific probes for each symbiont, nor did previous studies, which led to a single lineage sequence [16, 21] or unpublished data. Therefore, we cannot yet fully describe the distribution of either recovered *Zetaproteobacteria*-related MAGs.

We were nevertheless able to perform a phylogenomic analysis using 120 bacterial marker genes from our 49 MAGs, as well as 600 closely related genomes from GTDB, to infer how *Zetaproteobacteria* and other MAGs reconstructed in this study are distributed relative to known taxa (Additional File 9A). Both RB-MAG-00008 and TAG-MAG-00014 were found to be divergent, yet close relatives of *Ghiorsea bivora* isolated from TAG (Additional File 9B) [60]. RB_MAG_00008, estimated to have a genome size of 1,853,475 bp and 92.96% completion based on the presence of SCG genes [45], suggests an actual genome size of around 2 Mbp. TAG_MAG_00014, on the contrary, had a reconstructed genome size of 1,625,457 bp and 94.37% completion, suggesting an actual genome size of around 1.7 Mbp, that is, a slightly reduced and presumably streamlined genome. Nevertheless, this is to our knowledge the first study to report symbiotic *Zetaproteobacteria* genomes. Based on average nucleotide identity (ANI) values of 88.74% between the two newly reconstructed *Zetaproteobacteria* MAGs and less than 77% with their closest relatives *Ghiorsea bivora* from TAG, we propose that they belong to potentially novel species [61]. We suggest the names Candidatus *Ghiorsea rimicarensis* for RB_MAG_00008 and Candidatus *Ghiorsea crypta* for TAG_MAG_00014.

**Fig. 4:**
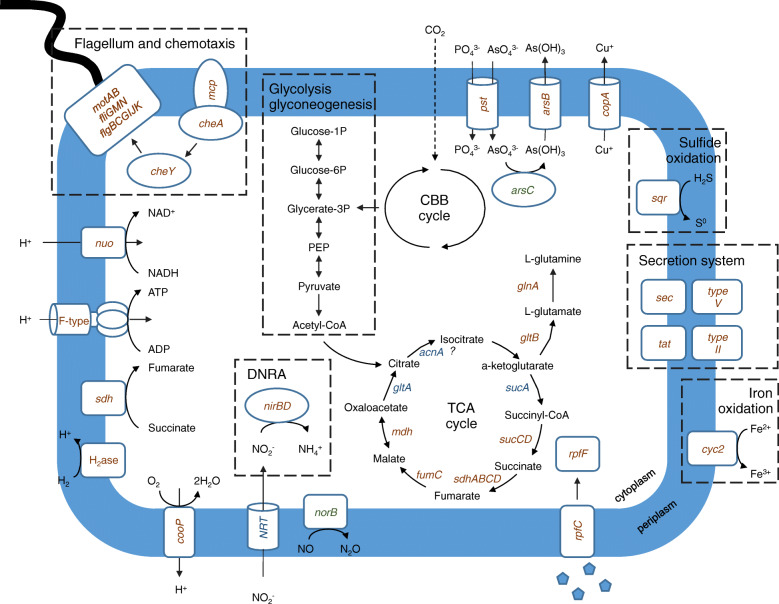
Schematic representation of the predicted metabolic potentials within *Zetaproteobacteria* MAGs: RB_MAG_00008 and TAG_MAG_00014. The potential metabolic traits are focused on carbon metabolism, nitrogen uptake, sulfur and iron oxidation, hydrogen utilization, flagellum, chemotaxis, and detoxification. Genes present in both MAGs are indicated in red; those present only in RB_MAG_00008 or TAG_MAG_00014 are indicated in blue and green, respectively. CBB, Calvin–Benson–Bassham; H_2_ase, Hydrogenase; PEP, phosphoenolpyruvate; SDH, succinate dehydrogenase

Like their closest cultivated counterpart, both of these *Zetaproteobacteria* MAGs have the complete set of genes to fix carbon though the CBB cycle ([57, 60], Fig. 4, Additional File 10), confirming the possibility of autotrophic carbon assimilation in these host-associated lineages. Both have the form II of ribulose-1,5-bisphosphate carboxylase/oxygenase (*cbbM* gene, Additional File 10), which is in agreement with their symbiotic life under external seawater flow with relatively low oxygen and high carbon dioxide content [14, 57]. In addition, although they are described as fully autotrophic bacteria under culturing approaches [55, 60], the presence of genes for putative heterotrophic behavior may suggest they are capable of mixotrophy, as already proposed by Singer and colleagues [62] for *M. profundus* PV1. These two *Zetaproteobacteria* MAGs share genes for formate/nitrite transporter (*focA*), di- and tricarboxylate transporters (*trk*), as well as putative tricarboxylic transport membrane protein (*tctA*) in RB_MAG_00018 (Additional File 11). These carbon compound transporters could be used by the symbionts to import small organic molecules. Notably, *focA* encodes the formate/nitrite transporter (FNT) family of integral membrane proteins that show a great specificity for small anions, formate, nitrite, hydrosulfide, and also larger organic acids [63, 64].

Both strains seem capable of using several electron donors. The detection, for the first time, of canonical *cyc2* iron oxidizing genes (Additional File 12) in both MAGs strengthens the possibility that these epibionts oxidize iron, as suggested by several authors [5, 8, 14, 21, 23, 24]. It is noteworthy that *cyc2* genes, and splits containing them, showed a slightly lower average coverage compared with the remaining *Zetaproteobacteria* genes for RB_MAG_00008 (Additional File 13A). These data could imply environmental populations with high levels of strain heterogeneity, as described in [65]. Furthermore, *Zetaproteobacteria* RB-MAG-0008 showed genes coding for multicopper oxidase (MCO), which have been shown as potential homologs for iron oxidation in several other taxa oxidizing iron [66].

Detection of sulfide:quinone oxidoreductase (*sqr*) in both bins could suggest the ability to use sulfur in addition to H_2_ and Fe(II), as shown for *G. bivora*, and further extend the potential metabolic repertoire of the *Zetaproteobacteria.* It is however of note that no other sulfur oxidation genes were retrieved. Cbb3-type cytochrome c oxidase encoding genes, involved in respiration, oxygen sensing, and detoxification [62], but also in aerobic neutrophilic Fe oxidation, and which are highly expressed in cultivated *Mariprofundus ferrooxydans* PV-1, were also found in both bins [67].

Nevertheless, we also observed striking differences between the two MAGs, such as the capacity for hydrogen oxidation using at least three NiFe hydrogenase coding genes (Hydrogenase I cytochrome b subunit (*hya*), NiFe bidirectional hydrogenase (*hox*), and NiFe- hydrogen uptake hydrogenase (*hup*) genes) that were detected in RB_MAG_00008 only (Additional File 10). This may contribute to niche partitioning between these symbionts, giving them more flexibility with regard to variable environmental conditions and avoiding potential competition.

Both MAGs show genes implied in dissimilatory nitrate reduction to ammonium (DNRA, using *nir* genes; Fig. 4 and Additional File 10), suggesting the potential ability for nitrogen acquisition, like their closest relative [60]. Glutamine synthetase and glutamate synthase enzymes were also encoded in both bins, suggesting that ammonia could be incorporated into amino acids, whether it comes from nitrate reduction or environmental uptake, and that the symbionts may, therefore, have the potential to recycle their host’s ammonium waste. Together with genes coding for other amino acids like threonine, these findings add to previous data showing that *Rimicaris* epibionts can synthesize amino acids. The recently reconstructed Candidatus *Desulfobulbus rimicarensis* from *Rimicaris* proved capable of synthesizing all 20 amino acids, with all the genes essential for amino acid biosynthesis present in the genome and expressed [19]. The presence of genes coding for thiamin (*thi*), biotin (*bio*), riboflavin (*rib*), and cobalamin (*cob*) biosynthesis also indicates that both MAGs probably have the ability to synthesize vitamins (Additional File 10 and 11).

Finally, we observed that numerous flagella biosynthesis encoding genes including hook-associated genes (*flgBCGIJK*), possibly from the same operon [68], as well as flagellar rotation genes (*motAB*), together with chemotaxis genes (*che*), were detected in both symbiotic bins. The presence of these genes suggests *Zetaproteobacteria* are able to actively move toward favorable environments, in agreement with host–symbiont recognition and colonization. The squid-vibrio symbiosis shows distinct flagellar functions ranging from swimming capacity to chemotaxis and host signaling and communication, which suggest essential and constitutive roles for these structures [69]. It is possible that flagella identified in free-living closest relative *G. bivora* [60] are also used for host–symbiont colonization.

Occurrence of genes coding for arsenic resistance (*arsBC*) proteins suggests the *Zetaproteobacteria* symbionts have the genetic potential for arsenic detoxification that might benefit the shrimps. Arsenic is considered as a toxic metalloid acting as a molecular analog of phosphate and glycerol, leading to metabolic damage (proteins, lipids, DNA breaks, and inhibition of DNA repair; for review, see [70]). In animals, intoxication leads to organ necrosis and brain damage.

We observed genes coding for twin-arginine translocation protein (*tat*), phosphate (*pst*), and copper transporters (*cop*) in each MAG, suggesting they can also transport proteins (including potential bacterial toxins and degradative enzymes such as proteases and lipases), ions, and metals. In addition, the presence of exopolyphosphatases (*ppx*) and polyphosphate kinase (*ppk*) in both MAGs suggests they are capable of producing polyphosphate, in agreement with previous observations [14, 21, 62]. In Fig. 4, we present a metabolic model highlighting the predicted metabolic potentials together with host–symbiont patterns for the zetaproteobacterial symbionts.

### Metabolically diverse *Rimicaris* epibionts

**Fig. 5:**
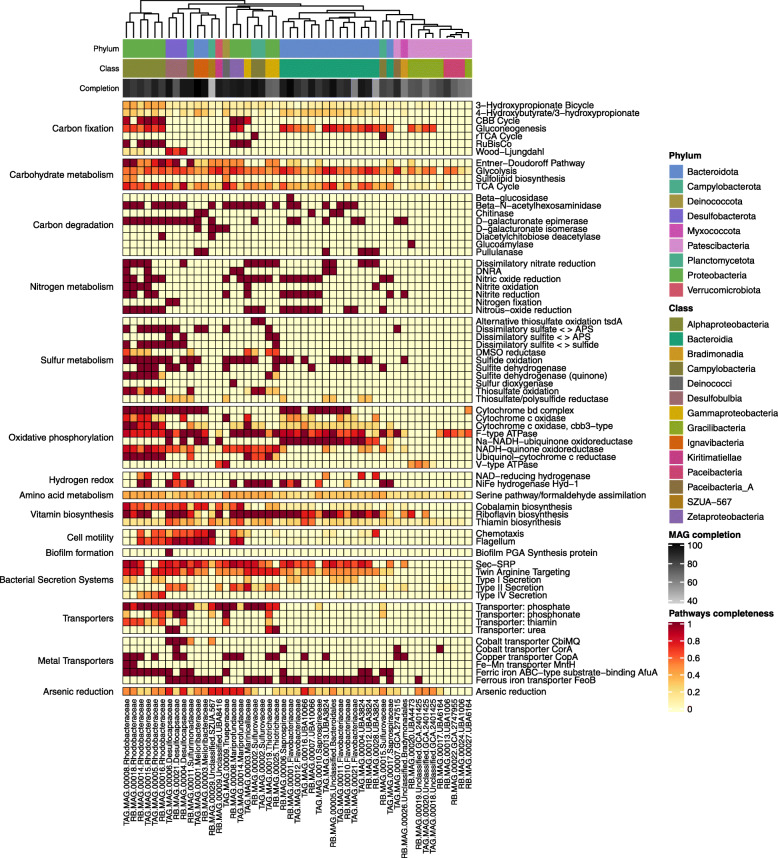
KEGG Decoder heat map based on KEGG annotation. The heat map represents metabolic pathway completeness of the MAGs based on the presence or absence of genes as determined by KEGG Decoder. The data represent the discussed pathways, all of which are shown in Additional File 15. The dendrogram at the top represents the similarity between the MAGs based on their metabolic pathways, using Euclidean distance and complete linkage clustering. Taxonomic affiliations at the class and phylum levels are indicated and represented by the different colors. NAD-reducing hydrogenase, *hox*HFUY; NiFe hydrogenase Hyd−1, *hya*ABC

Overall, we observed *Rimicaris* symbionts to have genomes with considerable functional diversity (Fig. 5; for details, see Additional File 14). The potential for autotrophic growth was observed in ten out of the 49 MAGs. Eight of those ten MAGs, affiliated to *Gamma*, *Zeta*, and *Alphaproteobacteria*, contained the complete set of genes for the CBB cycle. Our data confirmed that *Campylobacteria* MAGs were the only ones capable of mediating chemoautotrophy through the rTCA cycle. The presence of both rTCA and CBB cycles in the *Rimicaris* epibiont community has already been reported [16, 21, 47] and suggests that it might allow the consortium to switch cycles depending on the oxygen and carbon dioxide balance in the environment. The rTCA cycle, harboring oxygen-sensitive enzymes, is supposedly better adapted to more anoxic conditions combined with higher temperatures and is energetically more efficient than the CBB cycle [18, 71]. This property might partly explain the success of *Campylobacteria* in these environments. In addition, the *Desulfocapsaceae* MAGs from both Rainbow and TAG sites were shown to possess genes for the Wood-Ljungdahl pathway, allowing the use of hydrogen as an electron donor and carbon dioxide as both an electron acceptor and for biosynthesis, confirming the recent findings of [19].

Diverse energy sources potentially powering those symbioses were identified (Additional File 14). Genes related to sulfur metabolism, including *sox*, *sat*, *apr*, *dsr*, and *sqr*, were shown in 32 MAGs (that is, more than half of the population). Genes indicating the potential for hydrogen oxidation, including *hox* and *hyd* genes, were shown in 17 MAGs. This confirms the importance of H_2_ as an electron donor for the *Rimicaris* symbiosis, which probably contributes to its success at Rainbow where sulfide is lower. Finally, two *Zetaproteobacteria* MAGs may oxidize iron, as mentioned above. We observed 29 MAGs encoding the cbb3-type cytochrome c oxidase enzyme, used for sensing and respiration with oxygen as electron acceptor, but also for protection against oxidative stress. Four MAGs (belonging to *Rhodobacteraceae* and *Marinicellaceae* families) showed the possible use of an alternative electron acceptor to oxygen, such as nitrate as they encoded all the genes required for the complete reduction of nitrate to dinitrogen. Although the gene coding for nitrate reductase was lacking in *Campylobacteria*, possibly due to incomplete genome bins, the presence of three other denitrification genes suggests that they could also potentially use nitrate, as described in Jan et al. [21].

In addition, 30 other MAGs encoded some of the enzymes involved in nitrogen metabolism. Members of families like *Rhodobacteraceae* seemed to possess the capacity for denitrification, DNRA, and nitrification, which may provide them with greater metabolic flexibility compared with more specialist taxa. This seemingly redundant genetic potential for each cycle might indicate microniches, where each symbiont could perform part of a cycle relayed by another so as to perform complete pathways together. This is the case for carbon, as both autotrophy and heterotrophy fuel the host with diverse organic carbon [22], but also for nitrogen uptake and the sulfur cycle.

In addition, because the epibionts have the potential to synthesize and transport vitamins, such as thiamin, riboflavin, and cobalamin, the nutritional advantages for the host may go beyond a rich source of carbon and energy. The presence of genes for the biosynthesis of energy storage compounds, such as polyphosphate, supports previous results showing polyphosphate granules in the *R. exoculata* holobiont as well as in *M. ferrooxydans* cells [14, 62] and the genomic potential for their synthesis [21].

A total of 41 MAGs showed genes involved in arsenic reduction (*ars*RABC), which may be an environmental adaptation with the *ars* operon possibly being transferred among bacterial lineages. The presence of high concentrations of arsenic, highly correlated with zinc, was reported along the Mid-Atlantic Ridge [72]. Microorganisms having the *ars* operon, which can be extrachromosomal and subject to lateral gene transfer, are resistant and can cope with arsenic by reduction (*arsC*) and export (*arsB*, helped by *arsA*), which is regulated by arsenic level (*arsR*). Biofilms, chemotactism, flagellar synthesis, and quorum sensing are also enhanced by arsenic, which can be chelated by polysaccharides embedding cells in biofilms [73]. It is noteworthy that polymers were observed on MET on several *Rimicaris* samples [24]. In our model, *Campylobacteria*, *Gammaproteobacteria*, and now *Zetaproteobacteria* produce a biofilm, synthetize polysaccharides, and share *ars* genes. These lineages probably enter an arsenic cycle leading to chelation, which lowers potential impact on the host.

Finally, every 10 days, adult shrimp undergo a molt event, which necessitates continual and controlled microbiota colonization processes. Among them, secretion systems and biofilm formation are considered as central to host–symbiont recognition. In line with this, the majority of the MAGs [43] share characteristics of pathogens and beneficial microbes through genes encoding secretion systems (the general types II and IV and a striking number of twin targeting or Tat systems) and biofilm formation that probably facilitate their success at colonizing the host (detailed in Additional File 14).

### Niche partitioning in the *Rimicaris* holobiont

As for the *Zetaproteobacteria*, we observed genomic differences between closely related epibiotic strains that suggest niche partitioning is widespread in the *Rimicaris* holobiont. Genes for glycolysis, dissimilatory nitrate reduction, and thiosulfate oxidation were present in both RB_MAG_00025 and TAG_MAG_00019 abundant *Thiotrichaceae* symbiont populations. Nevertheless, enzymes for nitric oxide reduction, NiFe hydrogenase Hyd-1, and dissimilatory sulfate reduction were encoded only in TAG_MAG_00019, while those for dissimilatory sulfite reduction and sulfide oxidation was solely encoded in RB_MAG_00025. The observed functional differences between the closely related *Thiotrichaceae* MAGs could suggest some complementary rather than competitive strains and may explain their co-occurrence. It is also possible that one *Thiotrichaceae* strain performs some of the dissimilatory sulfate metabolic steps while the other covers the remaining ones. Likewise, both *Marinosulfonomonas* sp. RB_MAG_00014 and TAG_MAG_00015 have the potential for glycolysis, carbon fixation via the CBB cycle, and partial to complete reduction of nitrate to dinitrogen (N_2_), as well as sulfide and hydrogen oxidation (Fig. 5). Nevertheless, only TAG_MAG_00015 showed genes for both the complete oxidation of nitrate to N_2_ and DNRA. It should be noted that no *Marinosulfonomonas* sp. seemed able to utilize CH_4_, suggesting strains with different metabolic pathways than the ones described by Holmes et al. [74], putatively constrained by their association with animal hosts. Overall, we observed behind an apparent functional redundancy, a high symbiont strain diversity that possibly has important implications for the functioning of the complex *Rimicaris* symbiosis. These data are congruent with recent studies showing genomic heterogeneity in vent mussel symbiont populations that either possess or lack a key gene cluster, suggesting specialized rather than generalist symbionts [75, 76]. The high diversity of *Rimicaris* MAGs capable of sulfur oxidation is in agreement with previous work describing more than 16 different sulfur oxidizing strains in four *Bathymodiolus* species from the Mid-Atlantic Ridge, which showed a large adaptability to the holobiont in the vents [75]. These seemingly closely related strains were suggested to differ in key functions including the use of energy and nutrient sources, viral defense genes, and electron acceptors. These different studies posit that the costs for the maintenance of such symbiont diversity may be counterbalanced by the plasticity it offers, *i.e.*, a larger adaptability and resilience, especially in these unstable environments. In addition to obvious functional differences, it is likely that the symbionts have more subtle phenetic differences such as a better adaptation to temperature, chemicals, or pressure, allowing each of these strains to occupy and be adapted to different microniches. High levels of strain variability and numerous ortholog key proteins in vent-associated polychaete worm *Alvinella pompejana* were hypothesized as being each optimally adapted to thermal fluctuations within the worm’s habitat [77]. Similarly, Alcaide et al. [78] suggested that diverse carboxyl esterases of the gill-associated microbiota from *Rimicaris* may reflect distinct habitat-specific adaptations. Although it was not possible to determine whether geochemical or thermal fluctuations impose selection pressures on the epibiont community, this study adds to previous work showing that symbiont genetic diversity is more widespread than currently appreciated and that it might underpin ecosystem functioning and resilience in the highly dynamic hydrothermal vents.

## Conclusions

These data reveal a much more complex microbial consortium associated within *R. exoculata* than previously appreciated and highlight some generalized niche partitioning between symbionts. Our study stresses that the apparent functional redundancy at the genome-wide level between co-existing strains hides differences that may reflect distinct history traits. This may be the key to the success of this holobiont along the Mid-Atlantic Ridge, where it encounters contrasted habitats and shows itself capable of a great degree of connectivity [79]. Two zetaproteobacterial symbionts add to the metabolic catalog shared by the *Rimicaris* holobiont and to the repertoire of metabolic diversity and lifestyle of the *Zetaproteobacteria*. Taken together, our results reveal highly complex symbioses providing new insights into deep-sea ecosystem functioning and resilience to potential threats of anthropogenic or natural origin.

## Supplementary Information

**Additional file 1.** Details and scheme of the bioinformatics pipeline

**Additional file 2.** Quality trimming and filtering statistics for each individual, number of assembled contigs longer than 1 kbp for each site, and number of reads recruited by these contigs for each metagenome

**Additional file 3.** MAG collection, estimates of completion and redundancy calculated based on the occurrence of Single-copy Core Genes (SCG), number of contigs, total bin length, and taxonomic affiliation from TAG (above) and Rainbow (below) determined by GTDB-Tk

**Additional file 4.** GeTMM normalized counts of each MAG

**Additional file 5.** DESeq2 results table with baseMean, Log2FoldChange, lfcSE, stat, pvalue and padj for each of the 49 studied MAGs

**Additional file 6.** Differentially abundants MAGs between site identified through DESeq2. MAGs are indicated with the family they belong when they are affiliated until this level. Positive log2FoldChange indicate abundant taxa in Rainbow, negative log2FoldChange indicate abundant taxa in TAG. MAG are colored according to the phyla they belong. Cut off value for inclusion in the plot was 0.01 for padj and 2 for log2FoldChange absolute value. Dot sizes correspond to mean counts after normalization with GeTMM and DESeq2 (corresponding to baseMean in Additional file 5)

**Additional file 7.** PhyloFlash analysis results. Heatmap of taxonomic assignments (rows) for small-subunit rRNA reads in the six individual metagenomes (columns). The plot was generated using with the comparison script provided with phyloFlash. Color intensities represent the percentage of reads mapping to a given taxon, separated by prokaryotes (blue) and eukaryotes (red). Samples are clustered by their similarity in terms of taxonomic content and taxa are clustered by their co-occurrence across samples. Clustering are based on the euclidean distance and on the Ward's minimum variance method

**Additional file 8 **BLAST of 16S rRNA genes for *Zetaproteobacteria*. BLASTN search were performed between the 16S rRNA sequences affiliated to the *Zetaproteobacteria* assembled using SPAdes in phyloFlash and the 16S rRNA sequence retrieved from TAG_MAG_00014

**Additional file 9 A**. Maximum-likelihood tree based on concatenated marker proteins according to the GTDB-Tk genome phylogeny visualized using anvi’o. Tree includes 600 genomes from GTDB and 49 MAGs covering mostly unknown genera, highlighting the importance of lineages lacking representatives. A single Firmicutes was used to root the tree. The bars in the innermost circular layer show the phylum affiliation of each genome. The second layer represents the family affiliation. The third layer marks genomes as either MAGs from our study (49, black) or genomes from GTDB (grey). The outermost layer shows the genus affiliation (10) or the lack thereof (19) of our MAGs. Only the families and genera observed in the MAGs are shown. **B**. Zoom inset of the *Zetaproteobacteria* phylogenetic relationships visualized using FigTree. Nodes represented by a dot indicate a bootstrap value of 100; lower values are specified

**Additional file 10 **Key gene predictions from Zetaproteobacterial MAGs RB_MAG_00008 and TAG_MAG_00014 and free-living *Ghiorsea bivora* reference genome (NCBI accession number GCF_000744415.1) using RAST and FeGenie (indicated by a star). NA: “Not Available’

**Additional file 11.** Table containing the names of all genes found per MAG using KEGG and COG annotation

**Additional file 12 **Iron genes and gene clusters identified by FeGenie for the 49 MAGs and reference genomes *Ghiorsea bivora* and *Mariprofundus ferrooxydans* PV-1 (NCBI accession number GCF_000744415.1 and GCF_000153765.1). *Cyc2* genes were retrieved in both MAGs, confirming they have the potential to oxidize Fe (II)

**Additional file 13 **Differential coverage of contigs within *Zetaproteobacteria* bins. Static image from the anvi’o refine display for **A.** RB_MAG_00008 and **B.** TAG_MAG_00014. From inner to outer layers: clustering based on sequence composition and differential coverage with Euclidian distance and Ward clustering method, length layer (shows the actual length of a split), auxiliary layer with information about contigs stored in the contig database (GC-content), four view layers with information about MAGs across samples stored in the profile database (mean coverage), and Ribosomal RNA presence. Splits containing the *cyc2* genes are highlighted in red

**Additional file 14 **Analysis supplement: analysis details of metabolic potential among *Rimicaris* epibionts as depicted in Fig. 5

**Additional file 15 **KEGG Decoder heat map representing metabolic pathway completeness of the MAGs based on the presence or absence of genes as determined by KEGG Decoder. The dendrogram at the top represents the similarity between the MAGs based on their metabolic pathways, using Euclidean distance and complete linkage clustering. Taxonomic affiliations at the class and phylum levels are indicated and represented by the different colors. NAD-reducing hydrogenase: *hox*HFUY. NiFe hydrogenase Hyd−1: *hya*ABC

## Data Availability

All data generated or analyzed during this study are included in this published article (and its supplementary information files).
