# Correction to: Niche partitioning in the *Rimicaris exoculata* holobiont: the case of the first symbiotic *Zetaproteobacteria*

**DOI:** 10.1186/s40168-022-01257-4

**Published:** 2022-03-18

**Authors:** Marie-Anne Cambon-Bonavita, Johanne Aubé, Valérie Cueff-Gauchard, Julie Reveillaud

**Affiliations:** 1 Univ Brest, CNRS, IFREMER, Laboratoire de Microbiologie des Environnements Extrêmes, 29280 Plouzané, France; 2 MIVEGEC, Univ. Montpellier, INRAe, CNRS, IRD, Montpellier, France

**Correction to: Microbiome 9, 87 (2021)**

**https://doi.org/10.1186/s40168-021-01045-6**

Following publication of the original article [1], the authors reported an error in the sentence "It should be noted that no *Marinosulfonomonas* sp. seemed able to utilize CH4, suggesting strains with different metabolic pathways than the ones described by Holmes et al. [74], putatively constrained by their association with animal hosts" and would like to replace it by the following one **" It should be noted that no** ***Marinosulfonomonas*** **MAGs seemed able to utilize methanesulfonic acid (Additional File 11), suggesting strains with different metabolic pathways than the ones described by Holmes et al. [74], putatively constrained by their association with animal hosts".**

The authors apologize for any confusion this may have caused to the readers of the Microbiome Journal.
