# Supplementary material for: Niche partitioning in the Rimicaris exoculata holobiont: the case of the first symbiotic Zetaproteobacteria
Source: Microbiome. 2021 Apr 12;9:87. doi: 10.1186/s40168-021-01045-6 (PMC8042907; doi:10.1186/s40168-021-01045-6)
Supplement: Supplementary file 2 — Additional file 1. Details and scheme of the bioinformatics pipeline [file 40168_2021_1045_MOESM2_ESM.docx]

**Supplementary Information**

We generated an anvi’o contigs database from assembled contigs for each of the three co-assemblies, using the "anvi-gen-contigs-database" program, which uses Prodigal v2.6.3 [1] to identify ORFs and compute tetranucleotide frequency values in each contig. We built an Hidden Markov Model (HMM) profiling on resulting contig databases using the program "anvi-run-hmms", assigned gene functions using KEGG function with kofamscan [2] and COG function with “anvi-run-ncbi-cog”. We then used FeGenie to identify canonical iron genes [3]. We assigned taxonomy determined by the Genome Taxonomy Database (GTDB) using the "anvi-run-scg-taxonomy" program. We profiled the short reads from each of the six individual metagenomes recruited onto the contigs from the three co-assemblies using the "anvi-profile" program. We then merged resulting anvi’o profile databases for each sample using the "anvi-merge" program. We generated an automatic binning using CONCOCT v1.1.0 through the program "anvi-cluster-with-concoct" (using half of the estimated genome number found with the single-copy core genes) followed by manual refinement for each co-assembly. Since our study was based on single assemblies for each site, rather than on a co-assembly of all data together, we made a dereplication of metagenome-assembled genomes (MAGs) from the different studied sites with the aim of removing very closely related genomes and avoiding multiple high quality alignments during subsequent sequencing read mapping steps. We dereplicated MAGs with drep v 2.3.2 [4] using a coverage threshold ranging from 0.10 to 0.75 for comparison and an average nucleotide identity (ANI) > 98%. We performed a final mapping of all metagenomes on the dereplicated MAGs to calculate their mean coverage and detection.

**References**

1. Hyatt D, Chen GL, LoCascio PF, Land ML, Larimer FW, Hauser LJ. Prodigal: Prokaryotic gene recognition and translation initiation site identification. BMC Bioinformatics. 2010;11.

2. Aramaki T, Blanc-Mathieu R, Endo H, Ohkubo K, Kanehisa M, Goto S, et al. KofamKOALA: KEGG ortholog assignment based on profile HMM and adaptive score threshold. Bioinformatics. 2019;36:2251–2.

3. Garber AI, Nealson KH, Okamoto A, McAllister SM, Chan CS, Barco RA, et al. FeGenie: A Comprehensive Tool for the Identification of Iron Genes and Iron Gene Neighborhoods in Genome and Metagenome Assemblies. Front Microbiol. 2020;11:1–23.

4. Olm MR, Brown CT, Brooks B, Banfield JF. DRep: A tool for fast and accurate genomic comparisons that enables improved genome recovery from metagenomes through de-replication. ISME J 2017;11:2864–8.


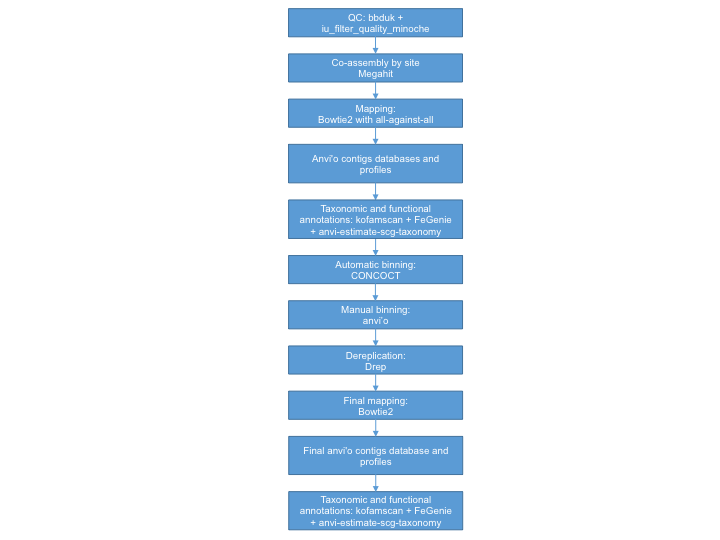


**Additional file 1.** Details and scheme of the bioinformatics pipeline. (DOCX 174 kb)
