# Supplementary material for: Niche partitioning in the Rimicaris exoculata holobiont: the case of the first symbiotic Zetaproteobacteria
Source: Microbiome. 2021 Apr 12;9:87. doi: 10.1186/s40168-021-01045-6 (PMC8042907; doi:10.1186/s40168-021-01045-6)
Supplement: Supplementary file 3 — Additional file 2. Quality trimming and filtering statistics for each individual, number of assembled contigs longer than 1 kbp for each site, and number of reads recruited by these contigs for each metagenome [file 40168_2021_1045_MOESM3_ESM.docx]

**Additional file 2.** Quality trimming and filtering statistics for each individual, number of assembled contigs longer than 1 kbp for each site, and number of reads recruited by these contigs for each metagenome. (XLSX 11 kb)
