# Supplementary material for: Niche partitioning in the Rimicaris exoculata holobiont: the case of the first symbiotic Zetaproteobacteria
Source: Microbiome. 2021 Apr 12;9:87. doi: 10.1186/s40168-021-01045-6 (PMC8042907; doi:10.1186/s40168-021-01045-6)
Supplement: Supplementary file 4 — Additional file 3. MAG collection, estimates of completion and redundancy calculated based on the occurrence of Single-copy Core Genes (SCG), number of contigs, total bin length, and taxonomic affiliation from TAG (above) and Rainbow (below) determined by GTDB-Tk [file 40168_2021_1045_MOESM4_ESM.docx]

**Additional file 3.** MAG collection, estimates of completion and redundancy calculated based on the occurrence of Single-copy Core Genes (SCG), number of contigs, total bin length, and taxonomic affiliation from TAG (above) and Rainbow (below) determined by the  Genome Taxonomy Database (GTDB). (XLSX 49 kb)
