# Supplementary material for: Niche partitioning in the Rimicaris exoculata holobiont: the case of the first symbiotic Zetaproteobacteria
Source: Microbiome. 2021 Apr 12;9:87. doi: 10.1186/s40168-021-01045-6 (PMC8042907; doi:10.1186/s40168-021-01045-6)
Supplement: Supplementary file 5 — Additional file 4. GeTMM normalized counts of each MAG [file 40168_2021_1045_MOESM5_ESM.docx]

Additional file 4. TMM normalized counts of each MAG. (XLS 34 kb)
