# Supplementary material for: Niche partitioning in the Rimicaris exoculata holobiont: the case of the first symbiotic Zetaproteobacteria
Source: Microbiome. 2021 Apr 12;9:87. doi: 10.1186/s40168-021-01045-6 (PMC8042907; doi:10.1186/s40168-021-01045-6)
Supplement: Supplementary file 7 — Additional file 6. Differentially abundants MAGs between site identified through DESeq2. MAGs are indicated with the family they belong when they are affiliated until this level. Positive log2FoldChange indicate abundant taxa in Rainbow, negative log2FoldChange indicate abundant taxa in TAG. MAG are colored according to the phyla they belong. Cut off value for inclusion in the plot was 0.01 for padj and 2 for log2FoldChange absolute value. Dot sizes correspond to mean counts after normalization with GeTMM and DESeq2 (corresponding to baseMean in Additional file 5) [file 40168_2021_1045_MOESM7_ESM.docx]

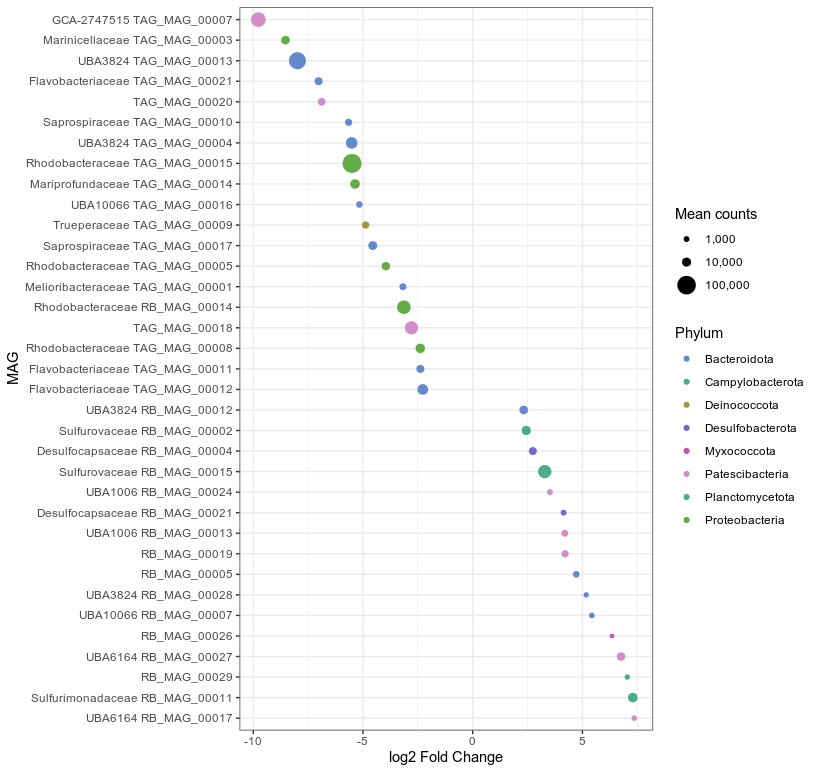
Additional file 6. Differentially abundants MAGs between site identified through DESeq2. MAGs are indicated with the family they belong when they are affiliated until this level. Positive log2FoldChange indicate abundant taxa in Rainbow, negative log2FoldChange indicate abundant taxa in TAG. MAG are colored according to the phyla they belong. Cut off value for inclusion in the plot was 0.01 for padj and 2 for log2FoldChange absolute value. Dot sizes correspond to mean counts after normalization with GeTMM and DESeq2 (corresponding to baseMean in Additional file 5). (PNG 99kb)
