# Supplementary material for: Niche partitioning in the Rimicaris exoculata holobiont: the case of the first symbiotic Zetaproteobacteria
Source: Microbiome. 2021 Apr 12;9:87. doi: 10.1186/s40168-021-01045-6 (PMC8042907; doi:10.1186/s40168-021-01045-6)
Supplement: Supplementary file 8 — Additional file 7. PhyloFlash analysis results. Heatmap of taxonomic assignments (rows) for small-subunit rRNA reads in the six individual metagenomes (columns). The plot was generated using with the comparison script provided with phyloFlash. Color intensities represent the percentage of reads mapping to a given taxon, separated by prokaryotes (blue) and eukaryotes (red). Samples are clustered by their similarity in terms of taxonomic content and taxa are clustered by their co-occurrence across samples. Clustering are based on the euclidean distance and on the Ward's minimum variance method [file 40168_2021_1045_MOESM8_ESM.docx]

**Additional File 7**. PhyloFlash analysis results. Heatmap of taxonomic assignments (rows) for small-subunit rRNA reads in the six individual metagenomes (columns). The plot was generated using with the comparison script provided with phyloFlash. Color intensities represent the percentage of reads mapping to a given taxon, separated by prokaryotes (blue) and eukaryotes (red). Samples are clustered by their similarity in terms of taxonomic content and taxa are clustered by their co-occurrence across samples. Clustering are based on the euclidean distance and on the Ward's minimum variance method. (PDF 39 kb)
