# Supplementary material for: Niche partitioning in the Rimicaris exoculata holobiont: the case of the first symbiotic Zetaproteobacteria
Source: Microbiome. 2021 Apr 12;9:87. doi: 10.1186/s40168-021-01045-6 (PMC8042907; doi:10.1186/s40168-021-01045-6)
Supplement: Supplementary file 9 — Additional file 8 BLAST of 16S rRNA genes for Zetaproteobacteria. BLASTN search were performed between the 16S rRNA sequences affiliated to the Zetaproteobacteria assembled using SPAdes in phyloFlash and the 16S rRNA sequence retrieved from TAG_MAG_00014 [file 40168_2021_1045_MOESM9_ESM.docx]

**Additional File 8**. BLAST of 16S rRNA genes for *Zetaproteobacteria*. BLASTN search were performed between the 16S rRNA sequences affiliated to the *Zetaproteobacteria* assembled using SPAdes in phyloFlash and the 16S rRNA sequence retrieved from TAG_MAG_00014 (DOC 128 kb).
