# Supplementary material for: Niche partitioning in the Rimicaris exoculata holobiont: the case of the first symbiotic Zetaproteobacteria
Source: Microbiome. 2021 Apr 12;9:87. doi: 10.1186/s40168-021-01045-6 (PMC8042907; doi:10.1186/s40168-021-01045-6)
Supplement: Supplementary file 10 — Additional file 9 A. Maximum-likelihood tree based on concatenated marker proteins according to the GTDB-Tk genome phylogeny visualized using anvi’o. Tree includes 600 genomes from GTDB and 49 MAGs covering mostly unknown genera, highlighting the importance of lineages lacking representatives. A single Firmicutes was used to root the tree. The bars in the innermost circular layer show the phylum affiliation of each genome. The second layer represents the family affiliation. The third layer marks genomes as either MAGs from our study (49, black) or genomes from GTDB (grey). The outermost layer shows the genus affiliation (10) or the lack thereof (19) of our MAGs. Only the families and genera observed in the MAGs are shown. B. Zoom inset of the Zetaproteobacteria phylogenetic relationships visualized using FigTree. Nodes represented by a dot indicate a bootstrap value of 100; lower values are specified [file 40168_2021_1045_MOESM10_ESM.docx]

**Additional File 9***:* **A**. Maximum-likelihood tree based on concatenated marker proteins according to the GTDB-Tk genome phylogeny visualized using anvi’o. Tree includes 600 genomes from GTDB and 49 MAGs covering mostly unknown genera, highlighting the importance of lineages lacking representatives. A single Firmicutes was used to root the tree. The bars in the innermost circular layer show the phylum affiliation of each genome. The second layer represents the family affiliation. The third layer marks genomes as either MAGs from our study (49, black) or genomes from GTDB (grey). The outermost layer shows the genus affiliation (10) or the lack thereof (19) of our MAGs. Only the families and genera observed in the MAGs are shown. **B**. Zoom inset of the *Zetaproteobacteria* phylogenetic relationships visualized using FigTree. Nodes represented by a dot indicate a bootstrap value of 100; lower values are specified. (PDF 87 kb).
