# Supplementary material for: Niche partitioning in the Rimicaris exoculata holobiont: the case of the first symbiotic Zetaproteobacteria
Source: Microbiome. 2021 Apr 12;9:87. doi: 10.1186/s40168-021-01045-6 (PMC8042907; doi:10.1186/s40168-021-01045-6)
Supplement: Supplementary file 11 — Additional file 10 Key gene predictions from Zetaproteobacterial MAGs RB_MAG_00008 and TAG_MAG_00014 and free-living Ghiorsea bivora reference genome (NCBI accession number GCF_000744415.1) using RAST and FeGenie (indicated by a star). NA: “Not Available’ [file 40168_2021_1045_MOESM11_ESM.docx]

**Additional File 10.** Key gene predictions from Zetaproteobacterial MAGs RB_MAG_00008 and TAG_MAG_00014 and free-living *Ghiorsea bivora* reference genome (NCBI accession number GCF_000744415.1) using RAST and FeGenie (indicated by a star). NA: “Not Available’. (XLSX 45 kb)
