# Supplementary material for: Niche partitioning in the Rimicaris exoculata holobiont: the case of the first symbiotic Zetaproteobacteria
Source: Microbiome. 2021 Apr 12;9:87. doi: 10.1186/s40168-021-01045-6 (PMC8042907; doi:10.1186/s40168-021-01045-6)
Supplement: Supplementary file 13 — Additional file 12 Iron genes and gene clusters identified by FeGenie for the 49 MAGs and reference genomes Ghiorsea bivora and Mariprofundus ferrooxydans PV-1 (NCBI accession number GCF_000744415.1 and GCF_000153765.1). Cyc2 genes were retrieved in both MAGs, confirming they have the potential to oxidize Fe (II) [file 40168_2021_1045_MOESM13_ESM.docx]

**Additional file 12.** Iron genes and gene clusters identified by FeGenie for the 49 MAGs and reference genomes *Ghiorsea bivora* and *Mariprofundus ferrooxydans* PV-1 (NCBI accession number GCF_000744415.1 and GCF_000153765.1). *Cyc2* genes were retrieved in both MAGs, confirming they have the potential to oxidize Fe (II) (XLSX 41 kb)
