# Supplementary material for: Niche partitioning in the Rimicaris exoculata holobiont: the case of the first symbiotic Zetaproteobacteria
Source: Microbiome. 2021 Apr 12;9:87. doi: 10.1186/s40168-021-01045-6 (PMC8042907; doi:10.1186/s40168-021-01045-6)
Supplement: Supplementary file 14 — Additional file 13 Differential coverage of contigs within Zetaproteobacteria bins. Static image from the anvi’o refine display for A. RB_MAG_00008 and B. TAG_MAG_00014. From inner to outer layers: clustering based on sequence composition and differential coverage with Euclidian distance and Ward clustering method, length layer (shows the actual length of a split), auxiliary layer with information about contigs stored in the contig database (GC-content), four view layers with information about MAGs across samples stored in the profile database (mean coverage), and Ribosomal RNA presence. Splits containing the cyc2 genes are highlighted in red [file 40168_2021_1045_MOESM14_ESM.docx]

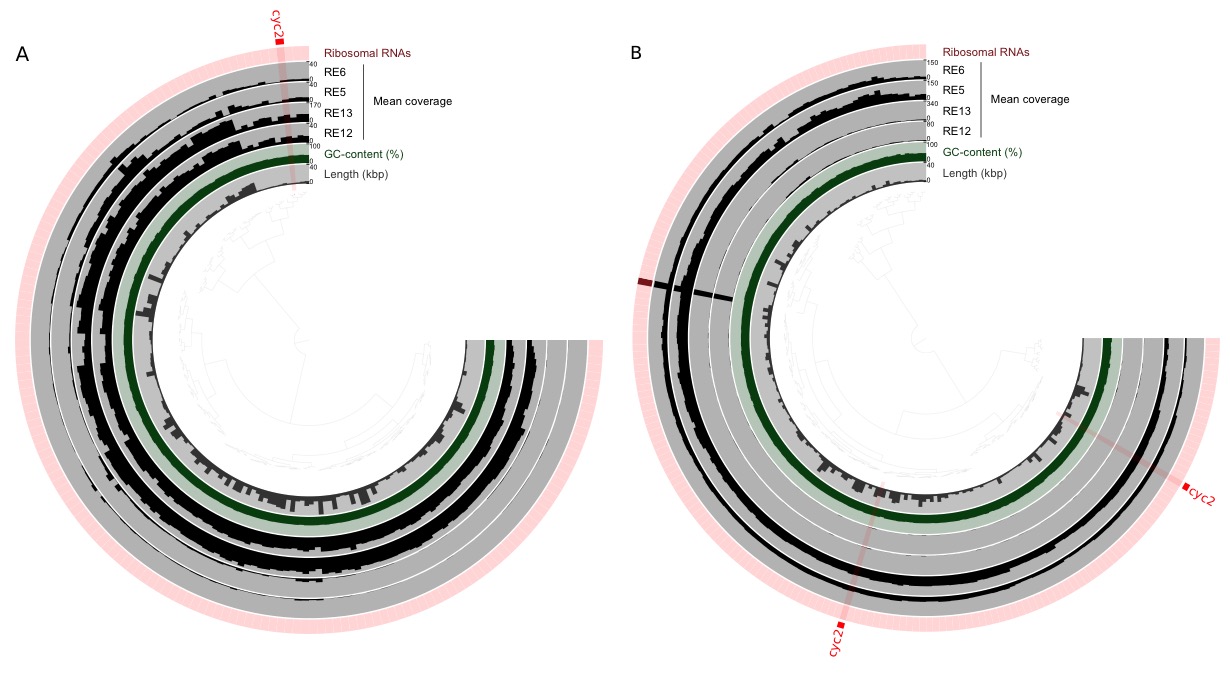


**Additional File 13.** Differential coverage of contigs within *Zetaproteobacteria* bins. Static image from the anvi’o refine display for **A.** RB_MAG_00008 and **B.** TAG_MAG_00014. From inner to outer layers: clustering based on sequence composition and differential coverage with Euclidian distance and Ward clustering method, length layer (shows the actual length of a split), auxiliary layer with information about contigs stored in the contig database (GC-content), four view layers with information about MAGs across samples stored in the profile database (mean coverage), and Ribosomal RNA presence. Splits containing the *cyc2* genes are highlighted in red. (PDF 129kb).
