# Supplementary material for: Niche partitioning in the Rimicaris exoculata holobiont: the case of the first symbiotic Zetaproteobacteria
Source: Microbiome. 2021 Apr 12;9:87. doi: 10.1186/s40168-021-01045-6 (PMC8042907; doi:10.1186/s40168-021-01045-6)
Supplement: Supplementary file 15 — Additional file 14 Analysis supplement: analysis details of metabolic potential among Rimicaris epibionts as depicted in Fig. 5 [file 40168_2021_1045_MOESM15_ESM.docx]

**Additional File 14**.Analysis supplement: analysis details of metabolic potential among *Rimicaris* epibionts as depicted in Figure 5. (DOCX 132kb)

Based on the 49 obtained MAGs, we identified the following capabilities in the shrimp microbiome.

**Autotrophic carbon fixation**

Eight MAGs belonging to *Gamma-*, *Zeta-,* and *Alphaproteobacteria* showed the capacity to fix carbon though the CBB cycle at both sites. In contrast, only two *Campylobacteria* *Sulfurovaceae* MAGs from Rainbow and TAG harbored the complete set of genes for the rTCA cycle. *Sulfurovaceae* TAG MAG 00002 did not contain rTCA cycle genes, nor did *Sulfurimonadaceae* RB MAG 00011. This is probably due to incomplete MAGs, as both lineages are known to share this cycle. The *Desulfocapsaceae* MAGs were the only ones harboring the Wood-Ljungdahl pathway genes for carbon assimilation at both TAG and Rainbow.

**Glycolysis, TCA cycle and carbon degradation**

Almost all the MAGs had the capacity for glycolysis and 43 showed the potential for the TCA cycle. We noted the potential for carbon degradation in 35 MAGs from both sites using glucoamylase (1 MAG), D-galacturonate epimerase (20 MAGs), D-galacturonate isomerase (4 MAGs), chitinase (5 MAGs), beta-N-acetylhexosaminidase (22 MAGs), beta-glucosidase (1 MAG) and pullulanase enzymes (6 MAGs). It is notable that chitinases could be used to graze on degrading chitin shrimp molts under heterotrophic metabolism, but bacteria can also secrete these enzymes for cell adhesion during pathogenesis and symbiosis [1].

**Sulfur metabolism**

***Dissimilatory sulfate reduction/sulfur oxidation***. The *apr*AB genes for adenosine phosphosulfate reductase and the *dsr* genes for dissimilatory sulfite reductase were found in four and nine MAGs, respectively. All these genes may also be involved in sulfur oxidation.

***SOX system***. Twelve MAGs from both TAG and Rainbow were observed to be capable of thiosulfate oxidation (using *sox* genes). The capacity for alternative thiosulfate oxidation (*tsdA* genes) was found in *Sulfurocaceae* MAGs from both sites. Thiosulfate polysulfide reductase (reverse), possibly converting thiosulfate into sulfite and hydrogen sulfide was identified in ten MAGs. We also identified the *sor* genes encoding sulfite dehydrogenase in ten MAGs and *soe* genes encoding sulfite:quinone oxidoreductase, both involved in sulfur oxidation, in eight MAGs. The capacity for sulfide oxidation using *sqr* genes was found in 25 MAGs. The capacity for potentially encoding DMSO reductase (using *dmsABC* genes) was identified in nine MAGs, although no phylogenetic analyses were performed.

**Iron metabolism**

Both *Zetaproteobacteria* MAGs showed the potential to oxidize Fe (II) using *Cyc2* genes [2,3].

**Hydrogen metabolism**

Hydrogen-metabolizing organisms use [NiFe]-hydrogenase to catalyze hydrogen oxidation. The *hoxHFUY* genes for NifFe NAD-reducing hydrogenase that couples reduction of NAD+ to the oxidation of hydrogen were identified in six MAGs from both sites and a total of 18 MAGs encoded the *hyaABC* genes for NiFe hydrogenase Hyd−1 (Hydrogenase 1).

**Nitrogen metabolism**

***Denitrification***. The potential for dissimilatory nitrate reduction using *nar* or *nap* genes was identified 15 MAGs from both sites. In addition, the capacity for the denitrification step of nitrite reduction requiring *nir* genes was observed in 14 MAGs, capacity for nitric oxide reduction requiring *nor* genes in 19 MAGs and capacity for nitrous oxide reduction requiring *nos* genes in 18 MAGs.

***DNRA***. Genes *nir* or *nrf* for dissimilatory nitrate reduction to ammonium (DNRA) were retrieved in six MAGs.

***Nitrification***. The key enzyme for nitrite oxidation, nitrite oxidoreductase (*nxr*), was identified in six MAGs. In addition, nitrogen fixation genes (*nif* genes) were retrieved in the two *Desulfocapsaceae* MAGs only, in agreement with the recent study of Jiang et al. (2020).

**Respiration and oxygen sensing**

Genes for cytochrome c oxidase (*cox*), ubiquinol cytochrome c reductase (*pet*;*fbc*), cytochrome c oxidase cbb3 type (*cco*) and cytochrome bd complex (*cyd*), which show different affinities for oxygen, were found in 18, 12, 29, and 22 MAGs from both sites, respectively.

**Symbiont-host colonization**

The potential capacity for biofilm exopolysaccharide PGA synthesis using *pgaABCD*, encoding an adhesin implicated in biofilm maintenance, was identified in one *Desulfocapsaceae* MAG only and the potential for sulfolipid biosynthesis was identified in one MAG from the *Trueperaceae* family. In addition, we observed an enrichment of genes coding for type I secretion systems within 11 MAGs from the *Rhodobacteraceae*, *Flavobacteriaceae*, and *Marinicellaceae* families. Type II encoding pili-related proteins were also observed in 18 MAGs. Overall, type I and type II mediate the secretion of a large variety of protein substrates (unfolded and folded) often associated with virulence [5,6], which could be used by symbionts to colonize hosts. Virulence factors such as hemolysins and chitinases exported by the type II secretion system have also been shown to be critical for initial establishment of symbiont *Aeromonas veronii* in the leech gut [7] or in the active invasion of the rice fungus *Rhizopus microsporus* by its endosymbionts *Burkholderia rhizoxinica* [8]. A type III secretion system was possibly identified in one *Mariprofundaceae* MAG, yet not all subunit genes were retrieved. These secretion systems are usually found in pathogenic bacteria, promoting the transfer of bacterial effector proteins to eukaryotic cells and promoting bacterial invasion and colonization [5,6]. Type IV secretion systems possibly involved in the dissemination of mobile genetic elements in addition to effector molecules were solely found in *Rhodobacteraceae* (4 MAGs) and *Flavobacteriaceae* (1 MAG) at both TAG and Rainbow. Type VI secretion systems were observed in the same *Rhodobacteraceae* MAGs as type IV. These secretion systems are reported to transfer toxic effector proteins into eukaryotic and prokaryotic target cells, to have an important role in pathogenesis and to defend bacteria against competing organisms [1]. The type IV secretion system also encodes pili that enable directional crawling (twitching motility), biofilm formation, and adhesion at the initial stages of colonization [9]. Twin arginine targeting (Tat) systems, another type of bacterial secretion system, documented to transport fully folded protein substrates and to be vital to bacteria [10], were identified in 36 MAGs.

**Metal transporters and detoxification**

Cobalt transporter genes *cbi* and *cor* were identified in five and four MAGs, respectively, belonging to *Desulfocapsaceae* and *Sulfurimonadaceae*. Copper transporters and ferrous iron transporters were found in 15 and 27 MAGs, respectively. Strikingly, a total of 41 MAGs showed the potential for dissimilatory arsenic reduction, with eight of them displaying most of the *ars* operon *arsRABC* (more than 75% pathway completeness). This latter allows the reduction (*arsC*), export (*arsB*, helped by *arsA*) and regulation of arsenic level (*arsR*). A potential role of detoxification through polymer chelation has also been reported [11].

**Vitamin biosynthesis and transporters**

A total of 48 MAGs showed the capacity to encode some of the genes required for thiamin biosynthesis and 37 could synthesize riboflavin and cobalamin, suggesting the importance of vitamin synthesis for the epibiont community. In addition, the presence of thiamin transporters (*tbp*A, *thi*P) in nine MAGs confirmed the capacity to export newly synthesized vitamins. The presence of genes coding for phosphate transporters (*pst*ABCS) in 24 MAGs across more than six families suggests the capacity and importance of phosphate uptake in the *Rimicaris* holobiont.

**Flagellum and chemotaxis**

Flagellum biosynthesis genes (represented by a subset of the *Fli, Flh* and *Flg* genes) were identified in 13 MAGs belonging to *Rhodobacteraceae* *Desulfocapsaceae*, *Melioribacteraceae*, *Sulfurimonadaceae* and *Mariprofundaceae*. The capacity for chemotaxis was identified in 19 MAGs belonging to the same families.

1. Yang Y, Sun J, Sun Y, Kwan YH, Wong WC, Zhang Y, et al. Genomic, transcriptomic, and proteomic insights into the symbiosis of deep-sea tubeworm holobionts. ISME J. 2020;14:135–50.
2. Barco RA, Emerson D, Sylvan JB, Orcutt BN, Jacobson Meyers ME, Ramírez GA, et al. New insight into microbial iron oxidation as revealed by the proteomic profile of an obligate iron-oxidizing chemolithoautotroph. Appl Environ Microbiol. 2015;81:5927–37.
3. Mori JF, Scott JJ, Hager KW, Moyer CL, Küsel K, Emerson D. Physiological and ecological implications of an iron- or hydrogen-oxidizing member of the Zetaproteobacteria, Ghiorsea bivora, gen. nov., sp. ISME J. 2017;11:2624–36.
4. Jiang L, Liu X, Dong C, Huang Z, Cambon-Bonavita M-A, Alain K, et al. Candidatus *Desulfobulbus rimicarensis*, an uncultivated deltaproteobacterial epibionts from the deep-sea hydrothermal vent shrimp *Rimicaris exoculata*. Appl Environ Microbiol. 2020;86(8):e02549-19.
5. Costa TRD, Felisberto-Rodrigues C, Meir A, Prevost MS, Redzej A, Trokter M, et al. Secretion systems in Gram-negative bacteria: Structural and mechanistic insights. Nat Rev Microbiol 2015;13:343–59.
6. Li Y, Liles MR, Halanych KM. Endosymbiont genomes yield clues of tubeworm success. ISME J. 2018;12:2785–95.
7. Maltz M, Graf J. The type II secretion system is essential for erythrocyte lysis and gut colonization by the leech digestive tract symbiont aeromonas veronii. Appl Environ Microbiol. 2011;77:597–603.
8. Moebius N, Üzüm Z, Dijksterhuis J, Lackner G, Hertweck C. Active invasion of bacteria into living fungal cells. Elife. 2014;3:e03007.
9. Grohmann E, Christie PJ, Waksman G, Backert S. Type IV secretion in Gram-negative and Gram-positive bacteria. Mol Microbiol. 2018;107(4):455-471.
10. Pickering BS, Oresnik IJ. The twin arginine transport system appears to be essential for viability in Sinorhizobium meliloti. J Bacteriol. 2010;192:5173–80.
11. Gupta P, Diwan B. Bacterial Exopolysaccharide mediated heavy metal removal: A Review on biosynthesis, mechanism and remediation strategies. Biotechnol Reports; 2017;13:58–71.
