# Supplementary material for: Niche partitioning in the Rimicaris exoculata holobiont: the case of the first symbiotic Zetaproteobacteria
Source: Microbiome. 2021 Apr 12;9:87. doi: 10.1186/s40168-021-01045-6 (PMC8042907; doi:10.1186/s40168-021-01045-6)
Supplement: Supplementary file 16 — Additional file 15 KEGG Decoder heat map representing metabolic pathway completeness of the MAGs based on the presence or absence of genes as determined by KEGG Decoder. The dendrogram at the top represents the similarity between the MAGs based on their metabolic pathways, using Euclidean distance and complete linkage clustering. Taxonomic affiliations at the class and phylum levels are indicated and represented by the different colors. NAD-reducing hydrogenase: hoxHFUY. NiFe hydrogenase Hyd−1: hyaABC [file 40168_2021_1045_MOESM16_ESM.docx]

**Additional File 15**. KEGG Decoder heat map representing metabolic pathway completeness of the MAGs based on the presence or absence of genes as determined by KEGG Decoder. The dendrogram at the top represents the similarity between the MAGs based on their metabolic pathways, using Euclidean distance and complete linkage clustering. Taxonomic affiliations at the class and phylum levels are indicated and represented by the different colors. NAD-reducing hydrogenase: *hox*HFUY. NiFe hydrogenase Hyd−1: *hya*ABC. (PDF 31kb).
